# Age-Related Homeostatic Plasticity at Rodent Neuromuscular Junctions

**DOI:** 10.3390/cells13201684

**Published:** 2024-10-11

**Authors:** Yizhi Li, Yomna Badawi, Stephen D. Meriney

**Affiliations:** Department of Neuroscience, University of Pittsburgh, Pittsburgh, PA 15260, USA; yil93@pitt.edu (Y.L.); y.badawi@pitt.edu (Y.B.)

**Keywords:** aging, neuromuscular junction, synaptic transmission, presynaptic homeostatic plasticity

## Abstract

Motor ability decline remains a major threat to the quality of life of the elderly. Although the later stages of aging co-exist with degenerative pathologies, the long process of aging is more complicated than a simple and gradual degeneration. To combat senescence and the associated late-stage degeneration of the neuromuscular system, it is imperative to examine changes that occur during the long process of aging. Prior to late-stage degeneration, age-induced changes in the neuromuscular system trigger homeostatic plasticity. This unique phenomenon may be important for the maintenance of the neuromuscular system during the early stages of aging. In this review, we will focus on age-induced changes in neurotransmission at the neuromuscular junction, providing the potential mechanisms responsible for these changes. The goal is to highlight these key elements and their role in regulating neurotransmission, facilitating future research efforts to combat late-stage degeneration in the neuromuscular system by preserving the functional and structural integrity of these elements prior to the late stage of aging.

## 1. Introduction

In 1920, only 1 in 20 people in the United States was aged 65 or beyond, while the 2020 census showed that this ratio had increased to 1 in 6 Americans [1]. Today, people are living longer, and ensuring a healthy late life has become an important medical focus as aging is often accompanied by a variety of challenging chronic conditions. Frailty in the elderly is one such chronic condition that can result in falls. Injuries resulting from falls severely affect the lives of the elderly: according to the Center for Disease Control and Prevention (CDC), falls are the leading cause of injury-related death among adults aged 65 and older, and the age-adjusted death rate due to falls has increased by 41% since 2012 [2]. The chance of falls doubles for individuals over the age of 75 [3]. Non-fatal falls in the older population create morbidity and a tremendous amount of financial and psychological burden caused by medical costs and a significant reduction in quality of life. Each year, the US spends more than $50 billion on non-fatal fall injuries [4]. This creates a strain on the medical care system, but more importantly, the lives of the elderly are significantly affected after a fall. Falls in the elderly can be attributed to losing muscle strength, developing balance deficits, or showing gait instability [5,6,7]. These phenomena are closely interlinked with the functions of the neuromuscular system. As the key component carrying out the motor command from the brain to the muscles, the neuromuscular system is one of the most vital systems keeping us mobile, or in the case of the diaphragm, keeping us alive and breathing. The neuromuscular system is not spared from degeneration in the process of aging. 

## 2. The Neuromuscular Junction and Its Role in Neuromuscular Aging

While sarcopenia, the loss of muscle mass, has been reported by many research groups as the cause of age-induced neuromuscular weakness [8], many research efforts have shown that restoring muscle mass in aged individuals has failed to completely restore motor functions [9,10,11,12,13,14]. Indeed, muscle is only one of the components of the neuromuscular system. Motor neurons and the connection between the motor nerve and muscle fibers, the neuromuscular junction (NMJ), are also involved in the changes associated with aging. A recent study looking at α-motor neurons in both monkeys and mice found that they do not atrophy in the spinal cord of either species [15]. However, these motor neurons are reported to receive less excitatory cholinergic and glutamatergic input at the spinal cord. In addition, the conduction velocity in the axons was reported to decrease with age [16,17], and this is hypothesized to be due to the loss of myelination (see review [18]). These changes in the motor neurons can affect the ability to initiate muscle contractions when they synapse onto the muscle fibers at the NMJs. While these are important aspects of the effect of aging on the neuromuscular system, we have chosen to focus this review on the NMJ itself. Because the neuromuscular junction functions as the site of communication between the motor nerve and the muscle fibers, it has been proposed as another focus in studying neuromuscular aging, and the term dynapenia (age-induced loss of muscle strength) has been introduced and incorporates multiple aspects of neuromuscular aging [10,11,19,20,21]. 

## 3. Components of the NMJ and the Importance of Its Homeostasis

The mammalian NMJ can be viewed as a three-part (tripartite) structure: the presynaptic motor nerve terminal, the postsynaptic muscle cell, and the terminal Schwann cells (tSCs). At the NMJ, active zones within the presynaptic motor nerve terminal are the sites where the neurotransmitter acetylcholine (ACh) is released. When ACh diffuses across the synaptic cleft and binds to ACh receptors (AChRs) on the muscle fibers, it can trigger the action potential to occur in the muscle fibers, which subsequently initiates muscle twitch [22,23]. More in-depth reviews of these postsynaptic structures can be found in reviews by C.R. Slater [24,25]. The tSCs function as an interactive third communicating component and help maintain the normal function of the NMJ [26,27,28,29]. Although these three parts are independent, their structures and functions are interlinked via many signaling molecules and proteins in the perisynaptic extracellular space. Signaling pathways between these three compartments modulate neurotransmission at the NMJ. One example of transsynaptic communication is the Agrin-LRP4 (low-density lipoprotein receptor-related protein 4)-MuSK (muscle-specific receptor tyrosine kinase) signaling pathway, which has been shown to be essential for both the formation and maintenance of the NMJ. Briefly, the release of agrin from the presynaptic nerve terminal binds to the LRP4 protein to form a protein complex with MuSK on the postsynaptic membrane, and this protein complex helps stabilize post-synaptic AChRs [30,31,32,33,34]. This signaling pathway serves as a tool to modulate the density and distribution of AChRs on the postsynaptic side, and it allows both the motor nerve and the muscle to fine-tune the communication between them. The tripartite structure of NMJ allows for complex signaling, plasticity, and adaptability, which are all key factors in maintaining proper structure and function at the NMJ. The complex interaction between these elements provides a framework within which to study the importance of homeostasis (or maintenance of normal function) regarding neurotransmission at the NMJ. In this review, we will highlight the effect of age on this homeostasis at the NMJ and subsequent functional changes. 

## 4. Measurements of Structure and Function of the NMJ

In rodents, the function of the NMJ is traditionally measured through either extracellular measures of the muscle compound muscle action potential (CMAP) or intracellular electrophysiological recordings of muscle membrane potential. Experimenters rely on measures of postsynaptic AChR-mediated events as proxies for the magnitude of chemical transmitter release from the nerve terminal. In these experiments, the changes in amplitude of the postsynaptic current through the AChR (endplate current; EPC) or postsynaptic membrane potential (endplate potential; EPP) that follows an action potential in the presynaptic nerve terminal are measured [35]. In addition, the subthreshold spontaneous release of transmitter resulting from the fusion of single synaptic vesicles with the presynaptic plasma membrane can also be recorded by measuring spontaneous events caused by the postsynaptic current through the AChR (miniature endplate current, mEPC) or muscle membrane potential changes (miniature endplate potential, mEPP) [36]. Based on the quantal hypothesis of transmitter release, transmitter is only released in packages (or quanta contained in synaptic vesicles) and the presynaptic action potential-evoked release of the neurotransmitter consists of the release of this transmitter from multiple synaptic vesicles over a short time period (several milliseconds). Using measures of postsynaptic endplate currents, we can estimate the number of synaptic vesicles that fuse with the presynaptic membrane after a presynaptic action potential (termed the quantal content) by dividing the EPC amplitude or integral by the mEPC amplitude or integral (or if measuring the postsynaptic membrane potential, the EPP amplitude by the mEPP amplitude) [37]. Quantal content (QC) can be used to estimate synaptic strength, and this varies across different specific neuromuscular synapses. For example, at the NMJ of the mouse epitrochleoanconeous (ETA) muscle, the average adult QC value is approximately 150 [38], while at the NMJs of the mouse tibialis anterior (TA) muscle, the value is about 80 [39,40]. Even though QC can vary at different NMJs, after development, the number of packages of transmitter released is always enough to bring the postsynaptic muscle cell to the threshold for firing an action potential and causing a muscle contraction. However, if QC is too low and does not reach the threshold, it can lead to ineffective communication between the motor nerve and the muscle fiber (failure to reach sufficient postsynaptic membrane depolarization to trigger an action potential and subsequent muscle twitch), causing functional deficits [41]. In addition to testing synaptic strength following a single presynaptic action potential, the motor nerve can be repetitively stimulated at a high frequency (tetanic stimulation or train stimulation) to measure the magnitude of transmitter release changes during repetitive stimulation over a short time period (usually several tens of milliseconds). A normal adult mouse neuromuscular synapse shows an initial, mild facilitation following the first few presynaptic action potentials in a train, followed by an eventual mild depression following additional action potentials in the train [42]. Taken together, these parameters are regularly used to gain insight into presynaptic function at the NMJ [38]. 

As to the structural integrity of the NMJ, researchers have relied on fluorescently conjugated toxins that label NMJ proteins or immunohistochemistry that targets specific proteins in the NMJ to visualize and quantify the NMJ structure and protein distribution. On the postsynaptic side of the synapse, the study of AChR density and distribution has been the main focus. Visualizing the location and distribution of the postsynaptic AChRs is performed using alpha-bungarotoxin (conjugated with a fluorophore) because of its high selectivity and affinity to AChRs, [43,44]. The very high density of AChRs within the synaptic cleft is critical to the strong and efficient communication between the motor nerve and the muscle fiber. When AChRs are compromised, as in the case of the neurological disease myasthenia gravis [45,46,47], neuromuscular transmission can be greatly reduced, leading to a failure of the presynaptic motor nerve action potential to result in a postsynaptic muscle cell action potential. 

**Figure 1:**
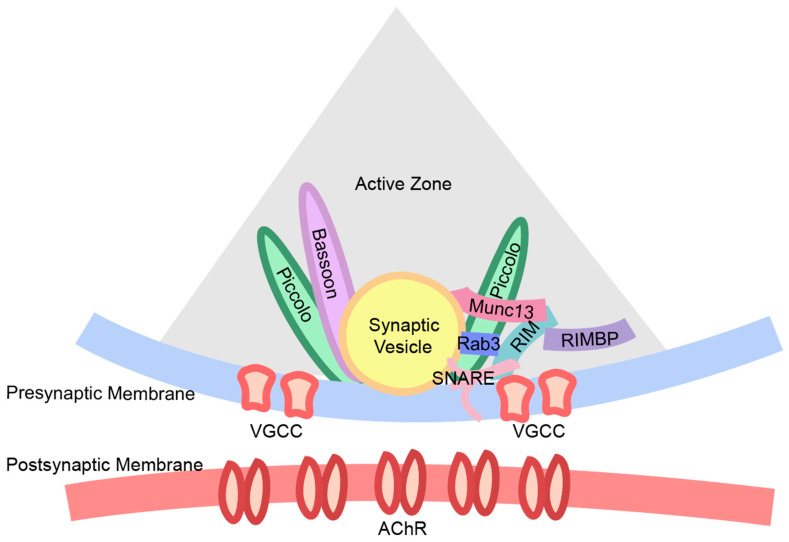
Diagram of a subset of proteins important for the control of neurotransmission at the NMJ, and their relative location within the active zone. Abbreviations: AChR—acetylcholine receptor; Munc13—mammalian uncoordinated-13; RIM—rab3 interacting molecule; RIMBP—rab3 interacting molecule binding protein; VGCC—voltage-gated calcium channel; SNARE—the soluble N-ethylmaleimide-sensitive factor attachment proteins receptors.

On the presynaptic side, many research efforts have focused on visualizing the density and distribution of a variety of motor nerve terminal proteins localized to transmitter release sites (active zones) and involved in action potential-triggered neurotransmitter release. In particular, the presynaptic voltage-gated calcium channels (VGCCs) gate the flux of calcium ions into motor nerve terminals, which trigger transmitter release from active zones [48,49,50], and their function, density, and distribution near an active zone strongly influence the probability of release [38,51,52,53,54]. This makes VGCC topography near the active zone a point of interest in elucidating the relationship between function and structure at the NMJ. In the active zone, there are many specialized proteins that control the release of neurotransmitters [55]. At mammalian NMJs, functions of active zone proteins like Munc13, RIM, RIM-BP, Piccolo, Bassoon, etc., have been shown to play a role in modulating neurotransmission [56] (see Figure 1). Bassoon and piccolo are cytomatrix proteins that organize and regulate the maintenance of active zones. As such, these two proteins are selectively expressed at vertebrate active zones [57,58,59,60] and are routinely used in immunohistochemistry experiments to reveal the location of the active zone. 

Labels for the presynaptic motor axons are also of interest to quantify the innervation of the neuromuscular junction. The motor axon is commonly visualized with an antibody against neurofilament, which is an axonal structural protein [61,62]. Some recent studies have found a possible link between the serum neurofilament level and neural degenerative diseases [63,64], making this protein a popular biomarker for axonal degeneration [65]. Lastly, to visualize the synaptic vesicles within the NMJ, researchers have often used antibodies against synaptic vesicle protein 2 [66] to quantify the extent of the nerve terminal that provides functional innervation. 

Taken together, proteins at the transmitter release sites on both pre- and postsynaptic sides of the NMJ influence neurotransmission. By monitoring the integrity of these markers at aged NMJs, we can quantify the effect of age on the NMJ. 

## 5. Aging and Plasticity

Aging at the NMJ is not only an important research topic as investigators aim to prevent age-induced muscle weakness but also an excellent model to study homeostatic plasticity at the NMJ. Aging includes physiological changes observed over the course of a lifetime. Although the later stages of aging co-exist with degenerative pathologies, the long process of aging is more complicated than a simple and gradual degeneration. Aging at the NMJ is a dynamic process over an extended period that includes changes due to homeostatic plasticity as the body attempts to compensate for age-induced reductions in neuromuscular function to preserve functional output. Age can often affect different features of the NMJ, and at different times, and it can become challenging to disentangle the underlying mechanisms when interpreting data collected over the aging time course across different laboratories. However, these plastic features at the NMJ have the potential to become targets for therapeutics or biomarkers for the biological age of the neuromuscular system and can help in understanding the process of neuromuscular aging. To provide context to the discussion of age-induced changes, we highlight the prior reports on the topic of age-induced homeostatic plasticity at the rodent NMJ.

## 6. Time-Dependent Changes in Synaptic Transmission 

Although reports on human NMJ aging also exist, it is difficult to establish a relatively comprehensive time course when constrained by the limited scope of experimental approaches to the human NMJ. Thus, this review will focus on changes and plasticity at the aging NMJ in rodent models. 

As mentioned above, the measurement of neuromuscular function is often through an assessment of synaptic strength, which is generally gauged by measuring the magnitude of neurotransmitter released following a presynaptic action potential. QC, which estimates the number of neurotransmitter packages (or quanta) released from the nerve terminal, serves as an estimate for synaptic strength. By comparing QC from different aging time points, one can determine the effect of age on synaptic transmission at the NMJ. In the case of an aged NMJ, one might assume that there would be a gradual decrease in neurotransmission with age. Although some studies indeed reported a decrease in QC [67,68], many other studies have shown the opposite [69,70,71], and some studies reported no changes in neurotransmission at the aged rodent NMJ [72]. These contradictory results could be due to the use of different species of animals, different muscle groups, different ages, or different sexes of animals. Therefore, to make it easier to sort these various aspects of experimental design in the evaluation of rodent NMJ aging studies, we have organized four tables outlining the effects of aging on the neuromuscular system; two that summarize studies on mice (Table 1 and Table 2) and two more that summarize studies on rats (Table 3 and Table 4).

## 7. Changes in Neurotransmission in Aged Mouse NMJ

At various time points between 25 and 30 months of age, the mouse NMJ displays either decreased, increased, or unchanged EPP or EPC amplitudes or QC when compared to NMJs of a young mouse (see Table 1). These conclusions are drawn from using different muscle groups and sometimes from using different strains of mice, leading to the hypothesis that different muscle groups adopt a different pace of aging. 

One study comparing 12–14-month-old mice with 24–28-month-old mice did not show any effect of aging on the magnitude of action potential-evoked transmitter release. At 28 months, the diaphragm (DIAPH) muscle in C57BL/6J mice showed increased EPC amplitude, but no significant change in QC was reported. In addition, they reported no changes in the magnitude of transmitter release in the extensor digitorum longus (EDL) muscle [72]. 

Other studies reported an increase in action potential-evoked transmitter release. These include a study on the mouse gluteus maximus (GM) muscle at 28 months, which showed increased QC [69], and a study on the Soleus (SOL) muscle, which reported an increase in QC [70]. 

Still others reported a decrease in action potential-evoked transmitter release, including a study at 29 months of age on the tibialis anterior (TA) muscle that reported decreased QC compared to their 23-month counterparts [67]. 

Lastly, sometimes, mixed results were obtained depending on the muscle studied. When comparing 11–13-month-old mice with either 29–30 or 34–35 months of age, the EDL and SOL showed increased EPP amplitudes, while DIAPH showed no change in the EPP amplitude [74]. 

However, since different specific time points were chosen for these studies, it is difficult to pinpoint exactly when neuromuscular aging starts and how it progresses over time. Our recently published report on age-induced changes in neurotransmission studied animals from 3–30 months of age, demonstrating that age-induced changes in neurotransmission at the ETA muscle of male mice follow a biphasic time course. This cross-sectional study showed an increase in QC from 19–24 months, followed by a decrease in QC from 25–30 months [73]. These findings demonstrate that the effect of age on neurotransmission at the mouse NMJ is dynamic. The results of this cross-sectional study can explain some of the discrepancies between previous studies when only studying one age time point (some reported an increase while others reported a decrease). Further, depending on the pace of aging for specific muscle groups, the effect that age can have on neurotransmission at the NMJ varies. Perhaps one of the most interesting observations from our cross-sectional analysis [73] was that before degenerative late-stage aging, there was a period where NMJs showed an increase in neurotransmission. We hypothesized that this increase in neurotransmission could be due to homeostatic plasticity, which may compensate for a reduced postsynaptic sensitivity that develops during early aging at the NMJ.

## 8. Changes in Morphology in Aged Mouse NMJs

One of the most consistent observations during aging is a fragmentation in the AChR distribution at the NMJ. A study looking at the sternocleidomastoid (SCM) muscle in 11-, 18-, and 22–26-month-old mice showed a significant increase in AChR fragmentation after 18 months [76] (See Table 2). Such an increase in AChR fragmentation can be seen in the ETA from 25–30-month-old mice [73]. Fragmented AChRs were also reported in a study using the DIAPH and EDL in 24–28-month-old mice [72]. The functional consequence of such fragmentation in aging may relate to alterations in postsynaptic sensitivity (which may trigger presynaptic homeostatic plasticity (PHP); see below). The physiological effects of these postsynaptic receptor changes require more dedicated studies. 

As for the total AChR area, a study that used electron microscopic images showed that 29–34-month-old mouse NMJs displayed a reduced AChR area and a reduced number of synaptic vesicles only in EDL, while SOL, GM, EDC, and DIAPH showed no significant changes in morphology [74]. More recently, Li et al. showed a decrease in the postsynaptic AChR area in the mouse ETA muscle from 19–24 months of age [73]. Such a decrease in the AChR area could lead to a decreased postsynaptic response after the evoked release from the motor nerve terminal, and this could trigger homeostatic plasticity to compensate for such a reduction in postsynaptic sensitivity [40]. 

Another mouse study using the SCM and transverse abdominus (TrA) muscles from 27-month-old animals found a significant decrease in the density of the presynaptic active zone-specific protein bassoon, suggesting a possible decrease in the density of synaptic vesicle release sites [79]. At 29 months, one ultrastructural study using EDL and SOL reported decreased nerve terminal areas, mitochondria count, and synaptic vesicle numbers [77]. Another study using 29-month-old mice showed that the SCM muscle displayed reduced VGCC and bassoon signal intensity levels [78]. These structural changes would be predicted to cause a disruption of neurotransmission at the mouse NMJ.

**Table 3:** Summary of prior reports of the effects of aging on the rat NMJ functions.

Parameter	Changes	Strain	Sex	Muscle	Young (Months)	Aged (Months)	Citation
Quantal Content	Increased	CFHB	M	DIAPH	N/A	1, 3, 7, 13	[71]
	Decreased	CFHB	M	DIAPH	N/A	1, 3, 7, 13	[71]
		Wistar	M	DIAPH	N/A	1, 4, 10, 20	[68]
Miniature End Plate Potential/Current	Increased mEPPs/mEPCs	Unknown	M	LA	2	30	[80]
	Decreased mEPPs/mEPCs	CFHB	M	DIAPH	N/A	1, 3, 7, 13	[71]
	No change in mEPPs/mEPCs	Wistar	M	DIAPH	N/A	1, 4, 10, 20	[68]
	Increased mEPPs frequency	CFHB	M	DIAPH	N/A	1, 3, 7, 13	[71]
		Sprague-Dawley	M	EDL	10	25–27	[81]
	Decreased mEPPs frequency	Unknown	M	LA	2	30	[80]
	No change in mEPPs frequency	Sprague-Dawley	M	DIAPH	10	25–27	[81]
		Sprague-Dawley	M	SOL	10	25–27	[81]
End Plate Potential/Current	Decreased	Wistar	M	DIAPH	N/A	1, 4, 10, 20	[68]

Abbreviations: diaphragm (DIAPH); extensor digitorum longus (EDL); end plate current (EPC); end plate potential (EPP); levator ani (LA); miniature end plate potential (mEPP); sternocleidomastoid (SCM); and soleus (SOL).

**Table 4:** Summary of prior reports of the effects of aging on the rat NMJ structures.

Parameter	Changes	Strain	Sex	Muscle	Young (Months)	Aged (Months)	Citation
Postsynaptic Acetylcholine Receptor	Increased AChR area	Fischer 344	M	DIAPH	6	24	[82]
		Fischer 344	M	SOL, EDL, DIAPH	N/A	10, 13, 16, 19, 22, 25, 28, 31	[83]
	Increased AChR fragmentation	Fischer 344	M	DIAPH	6	24	[82]
Presynaptic Nerve Terminal	Increased nerve terminal area	Fischer 344	M	DIAPH	6	24	[82]
	Increased branching number	Fischer 344	M	PM, SOL	9	20	[84]
		Fischer 344	M	SOL	7	23	[85]
		Fischer 344	M	DIAPH	6	24	[82]
		Fischer 344	M	PM, EDL	8	24	[86]
		Fischer 344	M	DIAPH	N/A	10, 13, 16, 19, 22, 25, 28, 31	[83]
	Decreased branching number	Fischer 344	M	EDL	N/A	10, 13, 16, 19, 22, 25, 28, 31	[83]
	No change in branching number	Fischer 344	M	SOL	N/A	10, 13, 16, 19, 22, 25, 28, 31	[83]
Presynaptic Structural Changes	Decreased BSN density	Sprague-Dawley	M	SCM	2	24	[87]

Abbreviations: Acetylcholine receptor (AChR); Bassoon (BSN); diaphragm (DIAPH); extensor digitorum longus (EDL); plantaris (PM); sternocleidomastoid (SCM); and soleus (SOL).

## 9. Changes in Neurotransmission in Aged Rat NMJ

One study on neurotransmission in rat DIAPH NMJs reported biphasic QC change (increasing until 7 months and then decreasing until 13 months) [71]. Another study in the DIAPH of rats aged 1, 4, 10, and 20 months reported decreased EPP amplitude at 20 months, while no significant change in mEPP amplitude was detected, contributing to a decreased QC at 20 months [68]. These studies (Table 3) can be useful to construct a time course of the neuromuscular aging profile of the diaphragm muscle in rats. It seems that the rat DIAPH also undergoes biphasic changes in terms of QC, albeit over a different time course than the ETA aging profile in mice. More studies on the effect of aging on QC in other muscles are needed to establish a timeline.

## 10. Changes in Morphology in Aged Rat NMJs

An early study on the morphology of NMJ aging using DIAPH, EDL, and SOL in rats at 10, 13, 16, 19, 22, 25, 28, and 31 months of age investigated a variety of morphological features and concluded that different rat muscles seem to age differently [83]. As mentioned above in the section focused on the mouse NMJ, this difference in the pace of aging in different muscles seems to be a theme in rodent species and possibly in mammals generally. 

One study using 20-month-old rat plantaris muscles (PMs) and SOL muscles demonstrated no effect of aging, except for increased presynaptic branching number and length [84]. The same conclusion was drawn when looking at SOL in 23-month-old rats [85] and in 24-month-old rats’ PM and SOL [86]. The increase in the axon branching number and length could indicate the sprouting of axons, which has been reported in a variety of neuromuscular diseases. Sprouting may represent a compensatory mechanism to rescue the loss of function. A detailed review of axonal sprouting at the NMJ can be found in a recent review [88]. 

At 24 months of age, one study found that the SCM muscle in rats showed decreased bassoon levels and increased AChR fragmentation (see Table 4). In this study, these features were ameliorated after 2 months of resistance training [87]. This study provides evidence for a potential therapeutic strategy directed against degeneration at aging NMJs. It also provides evidence that the system is plastic at 24 months of age, and the expression of some proteins, like bassoon and AChRs, is influenced by the usage of the muscles. 

One study using rat DIAPH at 24 months of age showed an increased AChR area [82]. This is the opposite of what has been reported in aged mouse NMJs. Although it could be a sign of postsynaptic compensation, the functional consequences of an increased AChR area on the postsynaptic side remain unknown.

Interestingly, the DIAPH has been reported in many studies [72,74,81,83] to be more resistant to age-related changes than other skeletal muscles (displaying fewer age-related changes). In interpreting these results, Smith [81] commented that constant usage during breathing might be a possible reason for the slower aging of the diaphragm as compared to a fast-twitch muscle like the EDL. In other words, because of the rhythmic breathing in mammalian animals, the diaphragm is constantly activated, and thus, the diaphragm appears to adopt a slower pace of aging. Although not directly contributing to neuromuscular weakness and falls, the diaphragm could be an interesting subject of study for its relative resistance to aging and the underlying mechanisms. Aside from the DIAPH, one study in mice found that several additional muscles (EOM, levator auris longus muscle, and frontalis) are also resistant to the effect of aging on NMJ morphology [75]. The exact reason behind this resistance is still unknown. One plausible explanation is the muscle fiber type composition. It has been reported that type I fibers are prone to inactivity and denervation while type II fibers are susceptible to aging, cancer, chronic heart diseases, and diabetes [89,90]. This observation can be complicated during the aging process as muscle fiber types have been reported to shift from type I to type II during aging [91]. Additional studies that focus on how fiber types may be altered in different muscles during the aging process are needed to decipher the underlying mechanisms that link aging resistance to muscle fiber type. 

Due to technical limitations, studies on the NMJ’s structure and function have not been conducted to directly correlate changes in neurotransmission with changes in morphology within the same synapse. Population studies are used to evaluate neurotransmission and structural changes at aged NMJs. Despite this population approach, many studies have attempted to elucidate the mechanisms behind structural changes at adult NMJs and the corresponding changes in neurotransmission. Below, we discuss some of the candidate mechanisms that may be responsible for the homeostatic plasticity previously described regarding the aging NMJ.

## 11. Presynaptic Homeostatic Plasticity (PHP) at the Aged NMJ

**Figure 2:**
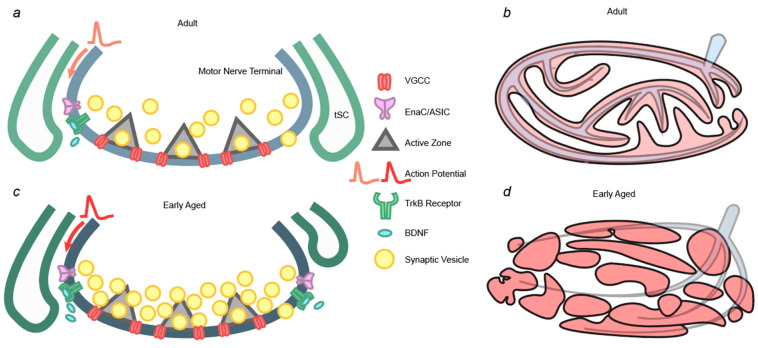
Summary of hypothesized mechanisms behind presynaptic homeostatic plasticity (PHP) at early-age neuromuscular junctions (NMJ). (**a**) A cross-sectional view of the presynaptic motor nerve terminal of an adult NMJ and the terminal Schwann cells (tSCs). Key elements that are proposed as potential mechanisms for PHP are highlighted in this diagram. (**b**) An *en face* view of typical postsynaptic acetylcholine receptors (AChR) at the adult NMJ. The innervating nerve is shown in grey. (**c**) An early-age presynaptic side of the NMJ and the tSCs. Key elements have either their quantity or morphology changed to reflect the increased neurotransmission at the early age stage of NMJ aging, including an increase in the size of the RRP, reduced tSC overlap with the NMJ, increase in the density and/or distribution of ENaCs and/or ASICs, increased BDNF and its TrkB receptor activity, and a broader AP waveform. (**d**) Increased postsynaptic AChR fragmentation, which may reduce postsynaptic sensitivity during early aging, triggering PHP. Abbreviations: tSCs—terminal Schwann cells; eNAc/ASIC—epithelial sodium channel/acid-sensing ion channel; TrkB—tyrosine kinase B; and BDNF—brain-derived neurotrophic factor; VGCC—voltage-gated calcium channels.

Transmitter release at the NMJ is highly plastic [92,93,94]. Homeostatic plasticity allows synapses to adjust their function to maintain a baseline of stable communication. Historically, studies of homeostatic plasticity have focused on changes in the number of postsynaptic receptors in response to a reduction or enhancement of central nervous system network activity [95,96]. More recently, PHP has been identified as a process that results in compensatory changes in the magnitude of neurotransmitter release in response to a perturbation of postsynaptic activity. Investigations of PHP have been most extensively carried out at the drosophila NMJ [97]. While there have been fewer studies of PHP at the mammalian NMJ, a homeostatic increase in transmitter release can occur in response to a partial block of postsynaptic receptors [98], and homeostatic plasticity is hypothesized to occur in the early phases of aging at the NMJ [99] to account for the increase in transmitter release that coincides with a decrease in AChR staining and the mEPP amplitude [73]. In fact, several of the reported changes in the magnitude of action potential-evoked transmitter release listed above (see “Change in mouse/rat neurotransmission” sections) might be triggered by plastic mechanisms that are initiated by alterations in postsynaptic receptor sensitivity. Reduced postsynaptic sensitivity can be evaluated in electrophysiological recordings by examining the mEPP or mEPC size: a smaller mEPP or mEPC amplitude is associated with reduced receptor sensitivity. Such reports on decreased mEPP or mEPC amplitudes have previously been reported by Jacob and Robbins [70]. Other reports found an increased mEPP or mEPC amplitude [67,72,80,100], and this could be the result of increased ACh sensitivity at the NMJs. Changes in postsynaptic sensitivity and presynaptic transmitter release have been reported to co-exist in both early- and late-stage aging of the NMJ. The postsynaptic receptor disruption and the increased neurotransmission in the early stage of aging are reminiscent of PHP, while the decreased neurotransmission in the late stage of aging could potentially be a result of PHP disruption. Below, we outline a variety of potential mechanisms that could cause PHP at the mouse NMJ based on a comparison of work in drosophila and rodent NMJs [101] (summarized in Figure 2). We highlighted these elements to facilitate further research efforts into these factors in both early-stage and late-stage aging of the NMJ, as they have the potential to be utilized as novel targets to combat dynapenia.

The first is an increase in the size of the readily releasable pool (RRP) of synaptic vesicles, which has been shown to underlie PHP at the mouse NMJ [98]. The number of synaptic vesicles released from an AZ is the product of the vesicle release probability (Pr) at each release site and the number of vesicles available for release (the RRP). At each mouse AZ, it was proposed that there are an average of two synaptic vesicles docked at each release site. The number of synaptic vesicles that occupy these release sites across the entire motor nerve terminal constitutes the RRP. The size of the RRP can therefore impact the number of synaptic vesicles released following an action potential stimulation, and this effect can be independent of other mechanisms that control the vesicle release probability (i.e., the density and distribution of VGCCs). RRP has been reported to be plastic in response to many perturbations at the NMJ [98,102,103,104,105]. Muller et al. [106] proposed that a change in RRP could be controlled by rab3 interaction molecules (RIMs), but the full extent of potential underlying mechanisms is still unclear. How RRP changes in the process of aging and the mechanisms responsible for these changes require further studies. 

A second potential mechanism that could underlie PHP at the mouse NMJ is a change in the density or distribution of the family of proteins that include the acid-sensing ion channels (ASICs) and epithelial Na+ channels (ENaCs) in the ENaCs/degenerin (Deg) family. These channels can depolarize the membrane potential on the presynaptic side, modulating neurotransmission. Zhu et al. showed that ASIC inhibitors block PHP triggered by a partial block of AChRs at the mouse NMJ [107]. Similarly, in drosophila, genes that encode ENaCs were shown to be necessary for PHP [108]. Interestingly, in a severe model of neurodegeneration in Drosophila, Orr et al. showed that ENaC channels could delay neurodegenerative pathophysiology, sustaining synaptic function, until more extensive degeneration had occurred [109]. This could be the reason behind the biphasic changes (early increase and later decrease) in neurotransmission at the NMJ at early or later aging time periods. 

A third mechanism that could cause PHP is a change in the presynaptic action potential (AP) waveform. The presynaptic AP, which is triggered by the opening of voltage-gated Na+ channels, followed by the subsequent opening of voltage-gated K^+^ channels, controls the opening of presynaptic VGCCs and the Ca^2+^ influx that will trigger transmitter release [110,111,112]. A prolonged presynaptic AP leads to a greater number of openings of VGCCs and a larger influx of Ca^2+^ during the evoked release event. This has been shown in experiments where 4-aminopyridine or 3,4-diaminopyridine (voltage-gated K^+^ channel blockers) were used to prolong the presynaptic AP, resulting in an increase in the amount of neurotransmitter released [113]. Another more recent study showed that in the central nervous system, the presynaptic AP is plastic, and the AP waveform changes in response to changing presynaptic calcium channel abundance and transmitter release probability. This homeostatic change in the presynaptic AP waveform is caused by changes in presynaptic Kv3 and Kv1 channels that alter AP waveform peak voltage [114]. In other words, the plasticity of the presynaptic AP waveform can serve as a modulator for synaptic function and is one component that can be responsible for PHP. If the properties of these K^+^ channels were affected by age, by altering the presynaptic AP waveform, we would expect a subsequent change in neurotransmitter release.

More than one of the mechanisms outlined above could underline the PHP associated with aging at the NMJ. For example, changes in ENaCs and/or ASICs could be upstream of changes in VGCCs and/or the RRP. In fact, ENaC or ASIC-induced depolarization and/or changes in VGCCs could increase presynaptic calcium and elevated calcium has been shown to increase the RRP [115]. Aside from changes in presynaptic VGCCs, Ca^2+^ is also proposed to influence PHP through the activity of the postsynaptic muscle cell. In this scenario, the calcium flux through activated postsynaptic nicotinic AChRs could trigger a positive feedback effect on the presynaptic motor nerve terminal, while the release of calcium from the sarcoplasmic reticulum followed by successful muscle contraction could serve as a negative feedback regulator [116]. Thus, in the case of insufficient activation of the muscle, the motor nerve terminal might receive only positive feedback to increase transmitter release, and this proposed mechanism is consistent with the increase in neurotransmission triggered by reduced AChRs in the early aging time period. What contributes to the loss of this communication and the subsequent reduction in neurotransmission in the later aging time period require further study. 

A fourth potential contributor to aged-associated PHP could be the terminal Schwann cells (tSCs). Although often overlooked, glial cells serve more functions than maintenance, and their contribution to the modulation of neurotransmission has recently been recognized. As the glial cells of the NMJ, tSCs are also known to regulate synaptic activity [117]. The first observation of such modulation reported a reduction in neurotransmitter release after increasing G-protein activity in the tSCs [118]. It was later discovered that tSCs regulate synaptic activity based on different types of motor nerve stimulation: tSCs induced synaptic depression after burst firing from the motor nerve, while long-lasting synaptic potentiation was observed after continuous stimulation [119], suggesting that tSCs have the ability to fine-tune the neurotransmission at the NMJ. The perturbation of tSCs and their function can lead to changes in neurotransmission in the process of aging. Indeed, it has previously been shown that more than 60% of the AChR area loses contact with tSCs [120] at 19 months, and the remaining 40% of the AChR area showed reduced capping surface (the surface area of AChRs that are covered by tSCs) [121,122]. Reduced tSC modulation at the early stage of NMJ aging can potentially contribute to increased neurotransmission. However, the exact role of tSCs in presynaptic transmission at different stages of NMJ aging is still poorly understood. As an important component of the NMJ, and a potential source of transmitter release [123], there should be further investigations into how tSCs change functionally in the process of aging, and subsequently, the effect of these changes on presynaptic neurotransmitter release. 

Lastly, the brain-derived neurotrophic factor (BDNF)–tropomyosin receptor kinase B (TrkB) pathway has been shown to modulate transmitter release at the NMJ [124], and this BDNF-TrkB pathway has been postulated to be a candidate mediator of age-induced changes [125,126,127,128]. In addition, it has been reported that a downstream target of the BDNF-TrkB pathway, the mammalian target of Rapamycin (mTOR), is involved in the aging process, including the neuromuscular system [129,130,131]. There have been numerous attempts at exogenously increasing the level of BDNF to induce neuronal growth in a variety of disease conditions, but those attempts have not succeeded, suggesting the rate-limiting factor might be changes in the TrkB receptor for BDNF [132,133,134,135,136,137,138,139]. TrkB receptors have two isoforms at the NMJ via alternative splicing: full-length TrkB (TrkB.FL) or truncated TrkB (TrkB.T1). TrkB.T1 lacks the intracellular tyrosine kinase domain, rendering it unable to phosphorylate presynaptic protein kinase C (PKC). It has been shown that PKC activity in the presynaptic nerve terminal can modulate ACh release by phosphorylating proteins such as Munc18-1 and SNAP-25 [124,140,141,142]. In this case, the ratio of TrkB.FL to TrkB.T1 can determine the amount of PKC activity, which can lead to an increase or decrease in ACh release on the presynaptic side of the NMJ. Theoretically, if an increase in TrkB/BDNF activity can be triggered by reduced postsynaptic receptor sensitivity, this homeostatic change could then lead to increased presynaptic neurotransmission, which has been shown in the early stage of aging. The decrease in the TrkB.FL-to-TrkB.T1 ratio at the NMJ has been reported in multiple neuromuscular diseases [143]. In addition, TrkB expression at the NMJ has been shown to decrease in aging [144]. These reports suggest that in late-stage aging, a decrease in the TrkB.FL-to-TrkB.T1 ratio could be one of the underlying mechanisms responsible for the decreased neurotransmitter release at the NMJ. The downstream effects important for neuronal function and survival are triggered by activating the BDNF–TrkB pathway (see review [145]). Thus, disruption of this pathway might not only affect transmitter release but also the maintenance of structural integrity of the synapse through important downstream pathways including mitogen-activated protein kinase (MAPK), phosphatidylinositol 3 kinase (PI3K)/mTOR, or phospholipase C (PLC), severely impacting these critical pathways at the NMJ. These downstream pathways are involved in protein translation at the synapse (for a detailed review, see [146]), and the perturbation of local protein translation at the nerve terminal can severely hinder neurotransmission. Loss of this pathway for survival and plasticity could be a potential mechanism behind the late-stage degeneration at the NMJ. Given the important role of the BDNF–TrkB pathway in maintaining synaptic function, the field would benefit from more dedicated studies focused on alterations in the BDNF–TrkB pathway during aging. Such studies may allow the identification of critical periods and targets for novel therapeutic interventions. 

Of course, age-induced homeostatic plasticity can also occur on the postsynaptic side of the NMJ. The many factors associated with aged-induced changes on the postsynaptic side of the neuromuscular synapse have recently been reviewed by Deschenes et al. [147]. 

Although the following have been reported as age-induced degenerative processes, they could also be targets for PHP at earlier stages of aging.

The first potential mechanism is changes in the number of functional release sites at the presynaptic terminal. According to quantal analysis [35,36,37,148], the magnitude of neurotransmission (M) depends on the number of functional release sites (N), the probability of transmitter release at each release site (Pr), and the impact of a single quantum (transmitter contained in a single synaptic vesicle) on the measured response (Q). These variables can be used to describe the magnitude of transmitter release by the equation M = N × Pr × Q. For example, we hypothesized that there is a reduction in the number of functional release sites (N) that might underlie the late-stage reduction in neurotransmission observed at 24–30 months of age in mice [73]. In support of this hypothesis, we showed that short-term synaptic plasticity at the mouse NMJ across many ages (3–30 months) did not significantly change. Because short-term synaptic plasticity is sensitive to the Pr at each release site (N), the average Pr of all release sites at aged NMJs is not likely to change over the aging time course [73]. Therefore, independent of a potential change in quantum size (Q), the driver behind any change (increase or decrease) in the magnitude of transmission could be the number of functional release sites, or active zones (N). Although there are no reports regarding a potential change in functional release sites in the early stage of NMJ aging, we did observe increased neurotransmission during this period. A hypothesized mechanism behind this plastic change in presynaptic transmitter release could be the larger number of functional release sites at the nerve terminal. To address these questions in various stages of NMJ aging, higher-resolution studies of the Pr from each AZ at a mouse NMJ are needed to examine, in detail, such hypothesized changes in functional release sites at the presynaptic terminal of aged NMJs. 

Another factor that can influence PHP is a change in the density or distribution of presynaptic VGCCs [149]. There has been a report showing that in 29-month-old mouse NMJs, there is significantly reduced P/Q type VGCC labeling [78]. The AZ function is extremely sensitive to subtle changes in the density and distribution of presynaptic VGCCs [52]. Thus, if age decreases the density and/or disrupts the distribution of presynaptic VGCCs at the NMJ, the transmitter release magnitude at AZs will be reduced as a result. In contrast, the upregulation of VGCC near the transmitter release sites can increase the evoked release, and this phenomenon could potentially be one of the underlying mechanisms behind the increased neurotransmission at the early stage of aging. Given the critical role that VGCCs play in neurotransmission at the nerve terminal, a better understanding of changes in the density or distribution of VGCCs at different stages of aging can provide context for the functional alterations observed.

## 12. Conclusions

Homeostatic plasticity is crucial for adaptability. As is also observed in many areas of the nervous system, NMJs are able to utilize plasticity to react to a variety of changes to maintain stable and efficient communication between the nerve and the muscle. Many features that are important for proper neurotransmission at the NMJ can be modified during aging. While we devote research efforts to the symptomatic and late stages of aging, we should also appreciate that prior to this symptomatic stage of aging, homeostatic plasticity may assist in maintaining the system. Understanding such intricate mechanisms can help us build a more comprehensive time course of neuromuscular aging, making the varied mechanisms underlying different stages of the aging process transparent. This detailed understanding would allow us to develop treatment strategies that are tailored to the specific needs of the neuromuscular system at different times. Perhaps even goals such as prolonging the pre-symptomatic stage of aging and working towards efficient prophylactic measures might be achievable. Another exciting potential for elucidating these homeostatic changes during aging is to use them as biomarkers for neuromuscular aging. In conclusion, this review has outlined research on age-induced changes at the neuromuscular junction with a focus on homeostatic plasticity, with the goal of highlighting the importance of these processes, which we hope will inspire additional research devoted to this subject.

## Figures and Tables

**Table 1:** Summary of prior reports on the effects of aging on the mouse NMJ functions.

Parameter	Changes	Strain	Sex	Muscle	Young (Months)	Aged (Months)	Citation
Quantal Content	Increased	C57/BL6	M	ETA	3–6	18–30	[73]
		C57BL/6NNia	M	GM	10	28	[69]
		CBF-1	M	SOL	4–8	25–30	[70]
	Decreased	C57/BL6	M	ETA	3–6	18–30	[73]
		C57BL/6J	M/F	TA	23	29	[67]
	No change	C57/BL6	M	ETA	3–6	18–30	[73]
		C57BL/6J	M	DIAPH	12–14	24–28	[72]
		C57BL/6J	M	EDL	12–14	24–28	[72]
Miniature End Plate Potential/Current	Increased mEPPs/mEPCs	C57BL/6NNia	M	GM	10	28	[69]
		C57BL/6J	M/F	TA	23	29	[67]
		CBF-1	M	SOL	4–8	25–30	[70]
		CBF-1	M	DIAPH	8–12	29–33	[74]
	Decreased mEPPs/mEPCs	C57/BL6	M	ETA	3–6	18–30	[73]
		C57BL/6NNia	M	GM	10	28	[69]
	No change in mEPPs/mEPCs	CBF-1	M	EDL, SOL, GM, EDC	8–12	29–33	[74]
	Increased mEPPs frequency	C57BL/6NNia	M	GM	10	28	[69]
	Decreased mEPPs frequency	C57BL/6NNia	M	GM	10	28	[69]
		C57BL/6J	M/F	TA	23	29	[67]
		CBF-1	M	SOL	4–8	25–30	[70]
End Plate Potential/ Current	Increased	C57/BL6	M	ETA	3–6	18–30	[73]
		C57BL/6J	M	DIAPH	12–14	24–28	[72]
		CBF-1	M	EDL, SOL	11–13	29–33	[74]
	Decreased	C57/BL6	M	ETA	3–6	18–30	[73]
	No change	C57BL/6J	M	EDL	12–14	24–28	[72]
		C57BL/6J	M/F	TA	23	29	[67]
		CBF-1	M	DIAPH	11–13	29–33	[74]
Input Resistance	Higher	CBF-1	M	EDL, SOL, GM, EDC	8–12	29–33	[74]
	No change	CBF-1	M	DIAPH	8–12	29–33	[74]

Abbreviations: diaphragm (DIAPH); extensor digitorum communis (EDC); extensor digitorum longus (EDL); epitrochleoanconeous (ETA); end plate current (EPC); end plate potential (EPP); gluteus maximus (GM); miniature end plate current (mEPC); miniature end plate potential (mEPP); sternocleidomastoid (SCM); soleus (SOL); and tibialis anterior (TA).

**Table 2:** Summary of prior reports on the effects of aging on the mouse NMJ structures.

Parameter	Changes	Strain	Sex	Muscle	Young (Months)	Aged (Months)	Citation
AChR	Decreased AChR area	C57/BL6	M	ETA	3–6	18–30	[73]
		CBF-1	M	EDL	11–13	29–33	[74]
	No change in AChR area	C57/BL6	M	ETA	3–6	18–30	[73]
		CBF-1	M	DIAPH, SOL, GM, EDC	11–13	29–33	[74]
		Thy1-XFP	N/A	EOM	4–5	22–28	[75]
	Increased AChR fragmentation	C57/BL6	M	ETA	3–6	18–30	[73]
		FP-Transgenic	M/F	SCM	2	11, 18, 22–26	[76]
		C57BL/6J	M	EDL	12–14	24–28	[72]
		Thy1-XFP	N/A	EDL	4–5	22–28	[75]
Presynaptic Nerve Terminal	Increased nerve terminal area	C57BL/6NNia	M	GM	10	28	[69]
		CBF-1	M	EDL, SOL	6	29	[77]
		Thy1-XFP	N/A	EDL	4–5	22–28	[75]
	Decreased nerve terminal area	CBF-1	M	EDL, SOL	6	29	[77]
	No change in nerve terminal area	C57/BL6	M	ETA	3–6	18–30	[73]
	Multiple Innervation	C57/BL6	M	ETA	3–6	18–30	[73]
		Thy1-XFP	N/A	EDL	4–5	22–28	[75]
	Denervation	C57/BL6	M	ETA	3–6	18–30	[73]
		Thy1-XFP	N/A	EDL	4–5	22–28	[75]
Presynaptic Structural Changes	Decreased VGCC intensity	C57BL/6NCr	F	SCM	8	29	[78]
	Decreased BSN density	C57/BL	M/F	SCM, TrA	1	27	[79]
		C57BL/6NCr	F	SCM	8	29	[78]
	No change in Piccolo density	C57BL/6NCr	F	SCM	8	29	[78]
	Decreased AZ density	C57/BL	M/F	SCM, TrA	1	27	[79]
	Decreased mitochondria count	CBF-1	M	EDL, SOL	6	29	[77]
	Decreased synaptic vesicle number	CBF-1	M	EDL, SOL	6	29	[77]
		CBF-1	M	EDL	11–13	30 or 34	[74]
	Increased smooth ER area	CBF-1	M	EDL, SOL	6	29	[77]

Abbreviations: Acetylcholine receptor (AChR); Active zone (AZ); Bassoon (BSN); diaphragm (DIAPH); extensor digitorum longus (EDL); extraocular muscles (EOM); epitrochleoanconeous (ETA); endoplasmic reticulum (ER); gluteus maximus (GM); sternocleidomastoid (SCM); soleus (SOL); transverse abdominus (TrA); and voltage-gated calcium channel (VGCC).

## Data Availability

The dataset is available upon request from the authors.
